# Crocin prevents acute angiotensin II-induced hypertension in anesthetized rats

**Published:** 2017

**Authors:** Mohammad Naser Shafei, Abdolali Faramarzi, Abolfazl Khajavi Rad, Akbar Anaeigoudari

**Affiliations:** 1 *Neurogenic Inflammation Research Center, Faculty of Medicine, Mashhad University of Medical Sciences, Mashhad, Iran *; 2 *Neurocognitive Research Center, Faculty of Medicine, Mashhad University of Medical Sciences, Mashhad, Iran *; 3 *Department of Physiology, Faculty of Medicine, Mashhad University of Medical Sciences, Mashhad, Iran *; 4 *Department of Physiology, School of Medicine, Jiroft University of Medical Sciences, Jiroft, Iran*

**Keywords:** Crocin, Hypertension, Heart rate, Mean arterial pressure, Angiotensin II

## Abstract

**Objective:**

Angiotensin II (Ang II), the main product of renin-angiotensin system (RAS) has a well-known role in cardiovascular regulation. Over-production of Ang II is one of the important underlying mechanisms of hypertension. In this study, the effect of crocin on cardiovascular responses in rats with acute hypertension induced by Ang II was evaluated.

**Materials and Methods:**

Rats were divided into six groups (n = 6): 1) Control: rats that received saline, 2) Ang II: rats that received Ang II (300 ng/kg) infused in two min, 3) Losartan (Los) + Ang II : rats that received Los (10 mg/kg, i.v) before Ang II, and 4-6) Crocin (Cro) + Ang II groups: rats that received three doses of crocin (50, 100 and 200 mg/kg, slow i.v) 10 min before Ang II. Femoral artery and vein were cannulated for recording of cardiovascular parameters and injection of drugs, respectively. Systolic blood pressure (SBP), mean arterial blood pressure (MAP) and heart rate (HR) were continuously recorded by power lab system. After injection of reagents and extracts, maximum changes (∆) of MAP, SBP and HR were recorded and compared with control group.

**Results:**

Ang II (300 ng/kg) increased maximal changes in MAP, SBP and HR compared to control group (p<0.001) and Los significantly attenuated these effects of Ang II (p<0.001). Maximal changes of MAP, SBP and HR induced by Ang II, were significantly attenuated by pretreatment with all doses of crocin (50,100 and 200) (p<0.05 and p<0.001). Also, changes of MAP, SBP and HR in Crocin + Ang II groups were significantly different from Los + Ang II group (p<0.05 and p<0. 01).

**Conclusion:**

Based on the effects of crocin on acute Ang II-induced hypertension, it is hypothesized that the cardiovascular improving effects of crocin may be mediated via suppressing of RAS.

## Introduction

Hypertension is one of the most important cardiovascular risk factors in all over the world. Hypertension can induce health-threatening disorders such as heart diseases, congestive heart failure, stroke, impaired vision and kidney dysfunction (Xie and Zhang, 2012 ▶). Although the exact mechanism(s) of hypertension has remained unknown, several factors such as increased activity of the sympathetic and renin angiotensin system (RAS) as well as disruption of local regulatory mechanisms have been reported to be involve in pathogenesis of this disorder (Veerasingham and Raizada, 2003 ▶). 

Renin- angiotensin system (RAS) has been reported to play an important role in cardiovascular regulation (Ibrahim 2006 ▶; Mehta and Griendling, 2007 ▶). The main active product of RAS, angiotensin II (Ang II), has been suggested to exert multiple physiological and pathological effects on cardiovascular system such as vasoconstriction, alteration in arterial baroreflex sensitivity, induction of vascular smooth muscle cell growth, stimulation of proto-oncogene expression, induction of myocardial hypertrophy and ventricular remodeling (Mehta and Griendling 2007 ▶; Rajagopalan et al., 1996 ▶; Taubman et al., 1989 ▶). The cardiovascular effects of Ang II were mostly mediated via AT1 receptor (Mehta and Griendling, 2007 ▶).

*Crocus sativus* (*C. sativus*), known as saffron, is a perennial stemless herb that is cultivated in Iran, especially South Khorasan (Srivastava et al., 2010 ▶). *C. sativus* possesses the dried red stigma with a small portion of the yellowish style attached to it (Hosseinzadeh and Talebzadeh, 2005 ▶). The value of *C. sativus* is attributed to the existence of three main constituents namely, crocin, picrocrocin, and safranal (Abe and Saito, 2000 ▶). Crocin is a water soluble carotenoid dye which gives food a golden-yellow tint. Based on previous studies, crocin has neuroprotective (Khazdair et al., 2015 ▶), smooth muscle relaxant effect (Mokhtari-Zaer et al., 2015 ▶), anti-tumor, radical scavenging, anti-hyperlipidemic and memory-improving effects (Razavi et al., 2013b ▶). Effect of crocin on blood pressure has been previously shown (Imenshahidi et al., 2010 ▶; Imenshahidi et al., 2014a ▶; Razavi et al., 2013a ▶). It has been reported that crocin dose-dependently attenuates hypertension induced by desoxycorticosterone acetate (DOCA) salt in rats (Imenshahidi et al., 2014b ▶). Crocin also improved cardiovascular toxicity caused by diazinon (DZN) via reducing oxidative stress in rats (Razavi et al., 2013a ▶). Although hypotensive effect of crocin on cardiovascular system has been shown but its exact mechanism is unknown. It seems that hypotensive effects of crocin partly were mediated via RAS inhibition. Therefore, in the present study, the effect of crocin on blood pressure and heart rate in rats with acute hypertension induced by Ang II was evaluated.

## Materials and Methods

**Experimental animals**

Thirty-six male Wistar rats (260±10 g) were kept in a colony room with 12 hr/12 hr light/dark cycle at 21 ± 2°C. The animals were freely allowed to have access to food and water. Animal examinations were carried out in accordance with procedures approved by the Committee on Animal Research of Mashhad University of Medical Sciences (Process Number 900559). 

**Drugs and reagents**

Ang II and urethane (Sigma, USA), losartan (Los, an angiotensin-II receptor blocker; as a gift from Darupaksh, I.R. Iran) and crocin were used. Crocin and losartan were dissolved in saline.

**Experimental groups**

Rats were randomly grouped (n = 6 in each group) into six groups as follow: 

Control group that received saline (intravenous (i.v)).

Ang II group that received Ang II (300 ng/kg, intravenously infused for 2 min).

Los group that received losartan (10 mg/kg, i.v) 2 min before injection of Ang II. 

Cro 50+Ang II that received crocin (50 mg/kg, i.v) and Ang II (300 ng/kg, i.v infusion for 2 min) 10 min after injection of crocin.

Cro 100 + Ang II that received crocin (100 mg/kg, i.v) and Ang II (300 ng/kg, i.v infusion for 2 min) 10 min after injection of crocin.

Cro 200 + Ang II that received crocin (200 mg/kg, i.v) and Ang II (300 ng/kg, i.v infusion for 2 min) 10 min after injection of crocin.

All doses were selected based on previous studies (Imenshahidi et al., 2010 ▶; Imenshahidi et al., 2014b ▶; Mahmoudabady et al., 2014 ▶)

**Experimental procedure**

Acute hypertension was induced by i.v infusion of Ang II according to previous studies (Mahmoudabady et al., 2014 ▶; Perez et al., 2010 ▶). In summary, Ang II (300 ng/kg) was intravenously infused for 2 min to achieve a high and sustained blood pressure (Bruner and Fink, 1985 ▶; Mahmoudabady et al., 2014 ▶). In crocin groups, three doses of crocin were i.v injected 10 min before Ang II. Volume of injection was 0.5 ml. Temperature was kept at 37.5°C, using a heating lamp. 

The animals were anesthetized with urethane (1.5 g/kg, i.p. and 0.7 g/kg as supplementary doses). After exposing the femoral artery, a polyethylene catheter- 50 filled with heparinized saline, was inserted in the artery and connected to a pressure transducer. The mean arterial pressure (MAP), systolic blood pressure (SBP) and heart rate (HR) were continuously recorded for 15 min using a power lab system (ID instrument, Australia) (Shafei et al., 2013 ▶). The femoral vain also cannulated for drug injection. 

**Data analysis**

All data were expressed as mean ± SEM. The maximum changes (∆) were obtained and compared with the control group. The data of maximal changes was compared using one way ANOVA followed by Tukey’s *post hoc* comparisons test. Differences were considered statistically significant when p<0.05.

## Results

**Effects of injection of saline on cardiovascular responses**

Baseline MAP, SBP and HR before injection of saline were recorded; then, saline was administered via the femoral vain. The results showed that administration of saline had no significant effect on MAP (before: 111.12±2.27 mmHg and after: 110.88±1.98 mmHg), SBP (before: 124.22±3.91 mmHg and after: 122.90±3.92 mmHg) and HR (before: 321.80±14.69 beats/min and after: 312.71 ± 15.7 beats/min).

**Effects of intravenous infusion of Ang II (300 ng/kg) on cardiovascular responses**

**Figure 1:**
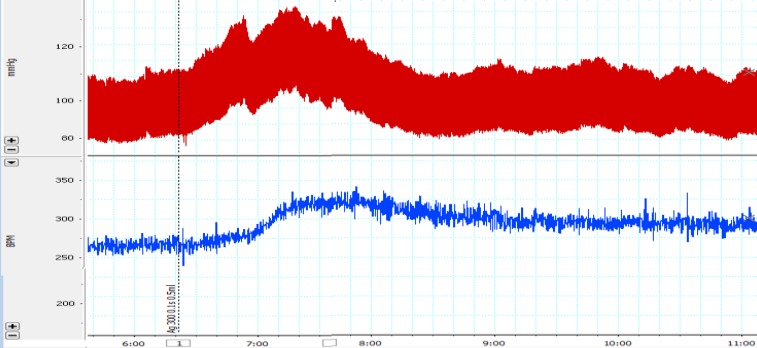
A typical record of blood pressure and heart rate in response to systemic infusion of Ang II (300 ng/kg). The vertical lines indicate the injection time

**Figure 2:**
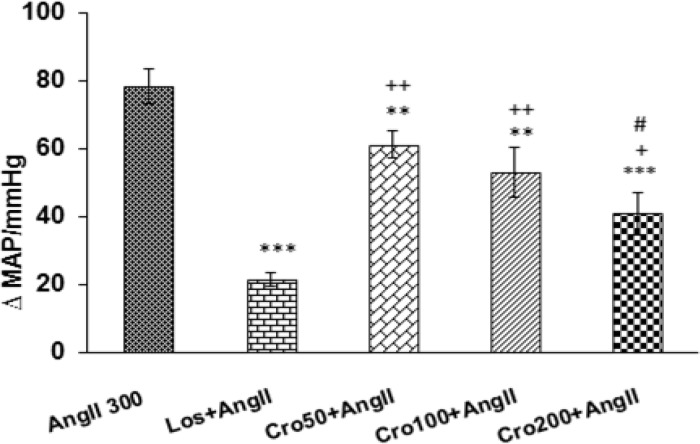
Maximal changes of systolic blood pressure (SBP) (A), mean arterial pressure (MAP) (B), and heart rate (HR) (C) in response to infusion of angiotensin II (Ang II 300 ng/kg) and Ang II+ Losartan (Los; 10 mg/kg). Data are expressed as mean ± SEM (n= 6 in each group). One way ANOVA with *post hoc* test used for statistical analysis. *** p<0.001 compared to control group, ^+++^ p<0.001 compared to Ang II 300 group and ^#^ p<0.05 and ^##^ p<0.01 compared to control group

In this study, Ang II (300ng/kg, i.v) was administered and cardiovascular responses were evaluated. Figure 1 shows a typical record of blood pressure and heart rate in response to Ang II injection. Maximal changes of SBP, MAP and HR are also shown in Figure 2. As shown in Figure 2, all parameters significantly increased compared to control group (p<0.001).

**Effect of intravenous injection of losartan on the cardiovascular responses in acute hypertension induced by angiotensin II**

 According to the results of the present study, pretreatment with losartan significantly reduced Ang II-induced MAP and SBP enhancement (p<0.001) (Figure 2). Pretreatment with losartan also diminished tachycardia induced by Ang II (p<0. 001) (Figure 2).

**Effect of pretreatment with different doses of crocin on the cardiovascular parameters in acute hypertension induced by angiotensin II**

**Figure 3:**
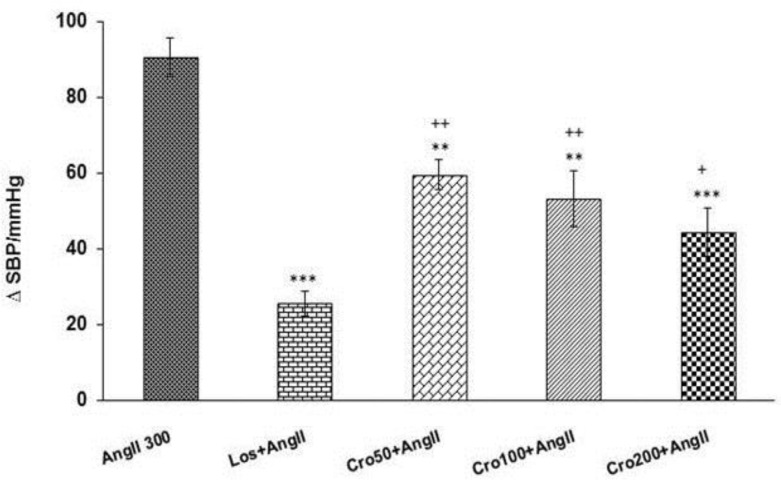
Maximal changes of mean arterial pressure (MAP) in response to intravenous injection of three doses of crocin (50,100 and 200 mg/kg) + (Ang II 300 ng/kg) compared to Ang II 300 group. Data are presented as mean ± SEM (n = 6 in each group). One way ANOVA with *post hoc* test used for statistical analysis. ** p<0.01 and *** p<0.001 compared to Ang II 300 group, ^++^ p<0.05 and ^++ ^p<0.01compared to Los + Ang II group, ^# ^p<0.05 compared to Cro 50 + Ang II and Cro100 + Ang II groups.

**Figure 4:**
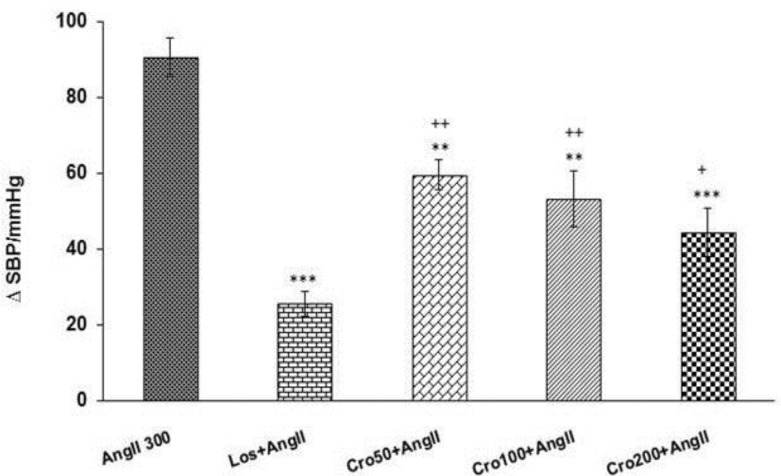
Maximal changes of systolic blood pressure (SBP) in response to intravenous injection of three doses of crocin (50,100 and 200 mg/kg) + angiotensin II (Ang II 300 ng/kg) compared to Ang II 300 group. Data are presented as mean ± SEM (n = 6 in each group). One way ANOVA with *Toky post hoc* test used for statistical analysis. ** p<0.01 and *** p<0.001 compared to Ang II 300 group, ^+^ p<0.05 and ^++^ p<0.01 compared to Los + Ang II group

The results of this research revealed that administration of crocin (50 and 100 and 200 mg/kg) before Ang II significantly attenuated the enhancement effects of Ang II on MAP and SBP compared to Ang II group (p<0.01 and p<0.001) (Figures 3 and 4). The results also indicated that MAP and SBP at all doses of crocin were significantly higher than Los+ Ang II group (p<0.05 and p<0.01) (Figures 3 and 4). In addition, MAP at the highest dose (200 mg/kg) of crocin was significantly lower than 50 and 100 mg/kg doses (p<0.05) (Figure 3).

**Figure 5:**
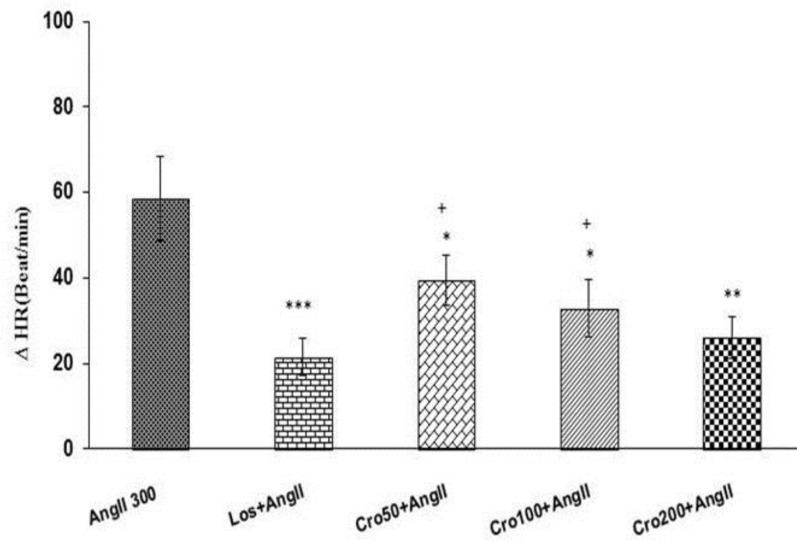
Maximal changes of heart rate (HR) in response to intravenous injection of three doses of crocin (50,100 and 200 mg/kg) + angiotensin II (Ang II 300 ng/kg) compared to Ang II 300 group. Data are presented as mean ± SEM (n = 6 in each group). * p<0.05, ** p<0.01 and *** p<0.001 compared to Ang II 300 group as well as ^+^ p<0.05 compared to Los + Ang II group

The results of current study also showed that all three doses of crocin (Cro 50+Ang II, Cro 100 + Ang II and Cro 200+Ang II) reduced changes of HR in comparison with Ang II group (p<0.05 and p<0.01) (Figure 5). In addition, changes of HR in Cro 50 + Ang II and Cro 100 + Ang II was significantly higher than changes in Los + Ang II group (p<0.05), but the in Cro 200 + Ang II group was not significant (Figure 5). There was also no significant different in changes of HR between all Cro + Ang II treated groups (Figure 5). 

## Discussion

The results of the current study showed that Ang II (300 ng/kg) administration significantly enhanced MAP, SBP and HR compared to control group and Los (an AT1 receptor blocker) significantly attenuated the cardiovascular effects of Ang II. In addition, pretreatment with crocin 50, 100 and 200 mg/kg were significantly decreased effects of Ang II on cardiovascular parameters. Moreover, the effects of the highest dose of crocin were comparable with those of losartan.

The Ang II, the main product of RAS, has been reported to play an important role in the cardiovascular system regulation (Mehta and Griendling 2007 ▶; Stegbauer et al., 2009 ▶). The acute effect of Ang II on the cardiovascular system is mostly mediated via vasoconstriction, increased sympathetic tone and arginine vasopressin release (Lavoie and Sigmund, 2003 ▶). 

Ang II has been also reported to contribute to the development of hypertension via enhancing the release of catecholamines from the adrenal gland and nerve terminals and increasing salt retention (Crowley et al., 2006 ▶). Previously, researchers have also propounded that Ang II-induced hypertension can take place through cellular events including promotion of phospholipase C and phosphatidylinositol hydrolysis, enhancement of intracellular free calcium concentration, activation of protein kinase C and decreased release of NO (Carey and Siragy 2003 ▶; Touyz 2003 ▶; Touyz and Schiffrin, 2000 ▶). In addition, Ang II has been reported to have a facilitating effect on Ca^2+^ mobilization from the sarcoplasmic reticulum (Mehta and Griendling, 2007 ▶). 

*C. sativus* and its active compound crocin have been reported to increase vasodilators such as NO (Mancini et al., 2014 ▶). He et al indicated that crocin can suppress Ca^2+^ release from the endoplasmic reticulum and inhibits influx of extracellular Ca^2+^ in aortic smooth muscle cells (He et al., 2004 ▶). Boskabady et al also showed that *C. sativus* exerts an inhibitory effect on calcium channels in pig’s isolated heart that is comparable with the effect of diltiazem on these channel (Boskabady et al., 2008 ▶). Considering these findings, it seems that restoring effect of crocin on Ang II- induced hypertension in current study is mediated through these pathways. 

In addition, growing evidence show that Ang II can influence blood pressure via stimulating the production of reactive oxygen species (ROS) and induction of oxidative stress (Laursen et al., 1997 ▶; Zimmerman et al., 2004 ▶). It has been indicated that increased levels of ROS followed by Ang II injection, result in vasoconstriction (Mollnau et al., 2002 ▶). In addition, Antioxidant effect of crocin has been documented (Asdaq and Inamdar 2010 ▶; Hosseinzadeh et al., 2009 ▶; Zheng et al., 2007 ▶). It has been reported that *C. sativus* and crocin reduce lipid peroxidation and enhance enzymatic and non- enzymatic antioxidant (Zheng et al., 2007 ▶). Therefore, it seems that a part of the improving effects of crocin on Ang II-induced hypertension may be mediated via its antioxidant effects. However, more investigations are needed to elucidate the exact mechanism(s). 

Losartan is a selective AT1 receptors antagonist which is demonstrated to induce experimental hypotension in experimental studies (Wong et al., 1990b ▶). Losartan has been reported to lower blood pressure in hypertensive rats (Wong et al., 1990a ▶). It has been suggested that this nonpeptide AT1 receptors antagonist might cause a renin-dependent hypotensive effect in patients with essential hypertension (Tsunoda et al., 1993 ▶). Losartan has been also proposed as an effective drug in regulating blood pressure and long-term renal damage in hypertensive patients (Patten and Abeywardena, 2017 ▶). The results of the present study also showed that administration of losartan before AngII attenuated the effect of Ang II on cardiovascular parameters. Therefore cardiovascular effect of Ang II is mostly mediated via AT1. Because crocin attenuate increased blood pressure and tachycardia induced by Ang II and these effects are comparable with losartan, it is possible that the effect of crocin on cardiovascular responses of Ang II is mediated via AT1 receptor. However, the exact mechanisms of these effects are not determined and needs further studies. 

In summary, regarding to modulatory effects of crocin on Ang II-induced hypertension, it seems that the inhibitory effect of crocin on the cardiovascular system was partly mediated via suppression of renin-angiotensin system.
